# The relative efficacy of topical non-steroidal anti-inflammatory drugs and capsaicin in osteoarthritis: a network meta-analysis of randomised controlled trials

**DOI:** 10.1016/j.joca.2018.08.008

**Published:** 2018-12

**Authors:** M.S.M. Persson, J. Stocks, D.A. Walsh, M. Doherty, W. Zhang

**Affiliations:** † Academic Rheumatology, Division of Rheumatology, Orthopaedics, and Dermatology, University of Nottingham, UK; ‡ Arthritis Research UK Pain Centre, UK

**Keywords:** Osteoarthritis, Topical, Capsaicin, Non-steroidal anti-inflammatory drugs (NSAIDs), Network, Meta-analysis

## Abstract

**Objective:**

To compare the efficacy of topical non-steroidal anti-inflammatory drugs (NSAIDs) with topical capsaicin for pain relief in osteoarthritis (OA).

**Design:**

A systematic literature search was conducted for randomised controlled trials (RCTs) examining any topical NSAID or capsaicin in OA. Pain relief at or nearest to 4 weeks was pooled using a random-effects network meta-analysis (NMA) in a Frequentist and Bayesian setting. Analysis was conducted for all trials and for trials using drugs listed as licensed for OA in the British National Formulary (BNF).

**Results:**

The trial network comprised 28 RCTs (7372 participants), of which 17 RCTs (3174 participants) were included in the as licensed analyses. No RCTs directly compared topical NSAIDs with capsaicin. Placebo was the only common comparator for topical NSAIDs and capsaicin. Frequentist and Bayesian effect size (ES) estimates were in agreement. Topical NSAIDs were statistically superior to placebo overall (ES 0.30, 95% confidence interval [CI] 0.19 to 0.41) and as licensed (ES 0.32, 95% CI 0.24 to 0.39). However, capsaicin was only statistically superior to placebo when used at licensed doses (ES 0.41, 95% CI 0.17 to 0.64). No significant differences were observed in pain relief between topical NSAIDs and capsaicin (overall: ES 0.04, 95% CI −0.26 to 0.33; as licensed: ES-0.09, 95% CI −0.34 to 0.16).

**Conclusions:**

Current evidence indicates that topical NSAIDs and capsaicin in licensed doses may be equally effective for pain relief in OA. Whether the equivalence varies between individuals remains unknown.

## Introduction

Osteoarthritis (OA) is a major cause of pain and disability for which two topical treatments are used: non-steroidal anti-inflammatory drugs (NSAIDs) and capsaicin^1^, ^2^, ^3^, ^4^, ^5^. Topical NSAIDs, such as ibuprofen and diclofenac, reversibly block the production of prostanoids, thereby reducing pain and inflammation^6^. Topical NSAIDs, alongside paracetamol, are recommended by the National Institute of Health and Care Excellence (NICE) as first line pharmacological treatments^1^. Over £32 million's worth of prescriptions of topical NSAIDs were dispensed in community pharmacies in England in 2016^7^. Topical NSAIDs are also freely available over-the-counter and are widely advertised to consumers. Meanwhile, capsaicin, the substance responsible for the warming spiciness of chili peppers, is primarily available on prescription in the UK. Almost 200,000 tubes of 0.025% capsaicin were dispensed in 2016, amounting to over £4 million^7^. Capsaicin is thought to cause defunctionalisation of spontaneously active peripheral nociceptors that otherwise maintain chronic pain conditions^8^.

Topical NSAIDs and capsaicin are applied directly to the skin over the painful joint and little to no active drug is absorbed into the bloodstream, resulting in their favourable safety profiles^8^, ^9^, ^10^. Topical administration therefore offers a safe and effective alternative to oral analgesics for people with just one or a few painful peripheral joints, especially for individuals with comorbidities, multiple medications, or those wishing to avoid tablets. The efficacy of topical NSAIDs and capsaicin in OA is documented^6^, ^11^, ^12^, ^13^, ^14^, however, no evidence for their relative efficacy is available so far to guide clinicians' prescribing practice. We therefore undertook the present network meta-analysis (NMA) to compare topical NSAIDs with capsaicin in people with symptomatic OA.

## Method

### Protocol and registration

This work forms part of a project examining the relative efficacy of topical NSAIDs and capsaicin in OA and neuropathic pain. The protocol is published^15^ and is also available on PROSPERO (2016:CRD42016035254).

### Eligibility criteria

Randomised controlled trials (RCTs) comparing any topical NSAID or capsaicin to placebo in participants with OA were included. No other comparators were included for this analysis and only placebo-controlled trials were examined. Participants with painful physician-diagnosed OA (clinical or radiographic) or chronic joint pain attributable to OA at any site (excluding the spine) were included. Spinal pain was excluded as it is difficult to differentiate between OA pain and back pain secondary to other aetiologies. Trials with pain due to multiple conditions were included if the data for OA could be extracted separately.

Trials had to be a minimum of 1 week duration and report pain outcomes. Full texts published in any language and at any date were considered.

### Identification and selection of trials

A search strategy, based on terms for (1) RCTs; (2) topical administration; (3) OA; and (4) capsaicin or NSAIDs, was created (Supplementary Information).

Medline, Embase, Allied and Complementary Medicine Database (AMED), Cumulative Index for Nursing and Allied Health Literature (CINAHL), Web of Science, and Cochrane library were searched up to 16/11/2015. The searches were updated on 10/01/2018. In addition, reference lists of included publications and meta-analyses in the area were searched for eligible trials.

Citations were exported to Endnote where duplicates were removed before titles, abstracts, and full texts were assessed for eligibility.

### Data collection and data items

The data were extracted independently by two authors (MSMP and JS) using a data extraction form created for this project. Publications in languages other than English were extracted by colleagues fluent in the language or using the Google Translate smart phone application. The following data were sought:

• Publication details: Author, journal, year
• Trial details: Country of study, trial funder, study design, blinding, setting, duration
• Participant details: Number of participants and withdrawals, age, gender distributions, body mass index, joint affected, method of diagnosing OA
• Intervention/placebo detail: Drug, formulation, dose/concentrations, frequency of application
• Endpoint: Pain scores

The primary end point was pain at or nearest to 4 weeks. Change from baseline pain scores (extracted or calculated) were used. If unavailable, endpoint pain scores or percent change from baseline were used. If pain was measured by more than one instrument in a study, the following hierarchy^16^, ^17^, ^18^ was used to extract pain outcome data: (1) visual analogue scale (VAS) global pain score; (2) categorical global pain score; (3) pain during activity, such as walking; (4) Western Ontario and McMaster Universities Osteoarthritis Index (WOMAC) pain subscale or pain subscale of other disease-specific composite tools; (5) Short Form-36 (SF-36) bodily pain subscale; (6) Health Assessment Questionnaire (HAQ) pain subscale, McGill pain questionnaire; (7) tenderness; (8) physician's assessment of pain. Where multiple concentrations of a study drug were examined within a study, they were combined as one prior to the effect size (ES) calculations for the overall analyses^19^.

### Network structure

A network diagram was plotted to illustrate the treatment nodes, direct comparisons, and indirect comparisons within the NMA.

### Risk of bias within and across studies

Risk of bias assessment was carried out independently by two authors (MSMP and JS) using a modified Cochrane Risk of Bias tool (Supplementary Material).

### Statistical analysis

Hedges' ES and corresponding standard error (SE) were calculated for each study. The estimates were combined using Frequentist and Bayesian random-effects NMAs. The Frequentist ES and associated 95% confidence interval (CI) were calculated. A Bayesian NMA was conducted using Markov chain Monte Carlo (MCMC) simulations. Non-informative prior distributions were set, normal likelihood distributions were assumed, and three Markov chains with different initial values (chosen arbitrarily) were run simultaneously. The model fit was deemed appropriate, the chain converged within 10,000 simulations, and a total of 20,000 simulations comprised the burn-in period. The subsequent 50,000 iterations were examined. The median and the 2.5th and 97.5th percentiles of the posterior distribution comprised the Bayesian ES and credible interval (CrI). The probability of each treatment being the best was calculated.

An overall analysis was conducted using all drug concentrations and topical formulations. Subgroup analysis was then conducted to examine topical NSAIDs and capsaicin used as recommended in the British National Formulary (BNF)^20^ (Supplementary Material). Trials were excluded from the *as licensed* analysis if they examined (1) topical NSAIDs not recommended in the BNF; (2) drugs used at concentrations lower than recommended; or (3) licensed drugs in formulations not in the recommended list. The *as licensed* analysis was conducted to guide clinical practice and inform decision-making based on the medications currently available to physicians.

The frequentist NMA was conducted in Stata (StataCorp. 2015. Stata Statistical Software: Release 15. College Station, TX: StataCorp LP) using the “network” command^21^. The Bayesian analyses were conducted in WinBUGs software (version 1.4.3, MRS Biostatistics Unit UK, 2007) using methods supplied by the NICE Decision Support Unit^22^.

## Results

### Study description

**Fig. 1:**
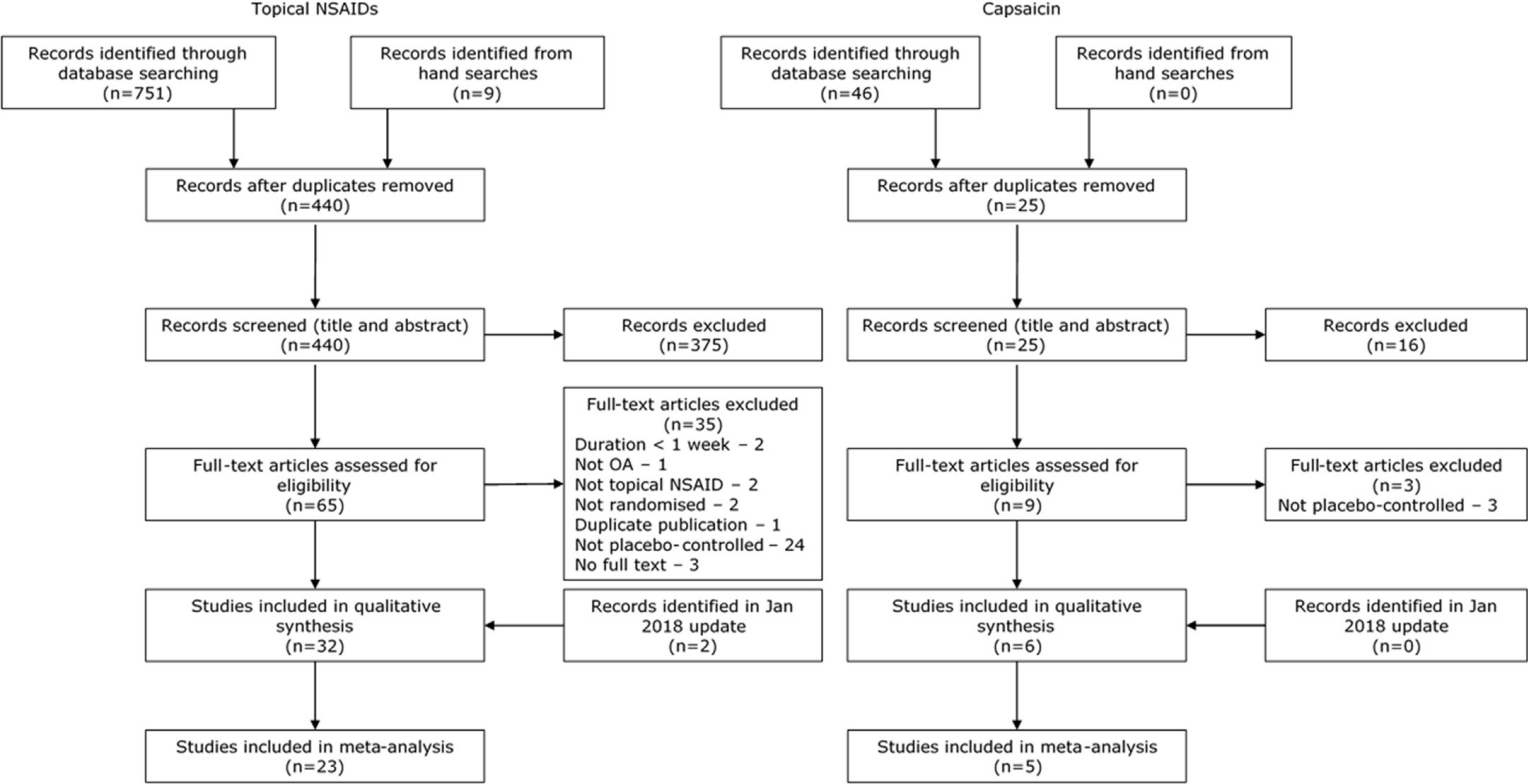
PRISMA flow diagram. Results of the systematic literature search for placebo-controlled trials of topical NSAIDs and capsaicin in OA.

The results of the literature search and reasons for exclusion from this meta-analysis are illustrated in Fig. 1. Topical NSAIDs were compared to placebo in 32 RCTs. Data were not available for extraction for nine of the studies^23^, ^24^, ^25^, ^26^, ^27^, ^28^, ^29^, ^30^, ^31^ and the remaining 23 studies (6957 participants)^32^, ^33^, ^34^, ^35^, ^36^, ^37^, ^38^, ^39^, ^40^, ^41^, ^42^, ^43^, ^44^, ^45^, ^46^, ^47^, ^48^, ^49^, ^50^, ^51^, ^52^, ^53^, ^54^ were included in the NMA. Of these, 13 trials^34^, ^35^, ^37^, ^38^, ^39^, ^40^, ^41^, ^42^, ^44^, ^46^, ^50^, ^52^, ^53^ used a topical NSAID at its recommended dose/formulation and were included in the *as licensed* analysis. Six placebo-controlled RCTs examining capsaicin were identified, of which five (415 participants)^55^, ^56^, ^57^, ^58^, ^59^ were included in the NMA. Data from the sixth study^60^ were not available for extraction. Four trials^56^, ^57^, ^58^, ^59^ used 0.025% capsaicin four times per day, as recommended in the BNF.

All trials were described as double-blinded and all but one^55^ were of parallel design. Data from the first period were extracted for the crossover trial. One publication was in Korean^48^ and the remainder were in English. 24 trials were limited to participants with knee OA, two to hand OA^34^, ^57^, and the two remaining trials^56^, ^58^ included OA at multiple sites (hand, wrist, elbow, shoulder, hip, knee, and ankle OA).

### Risk of bias

**Fig. 2:**
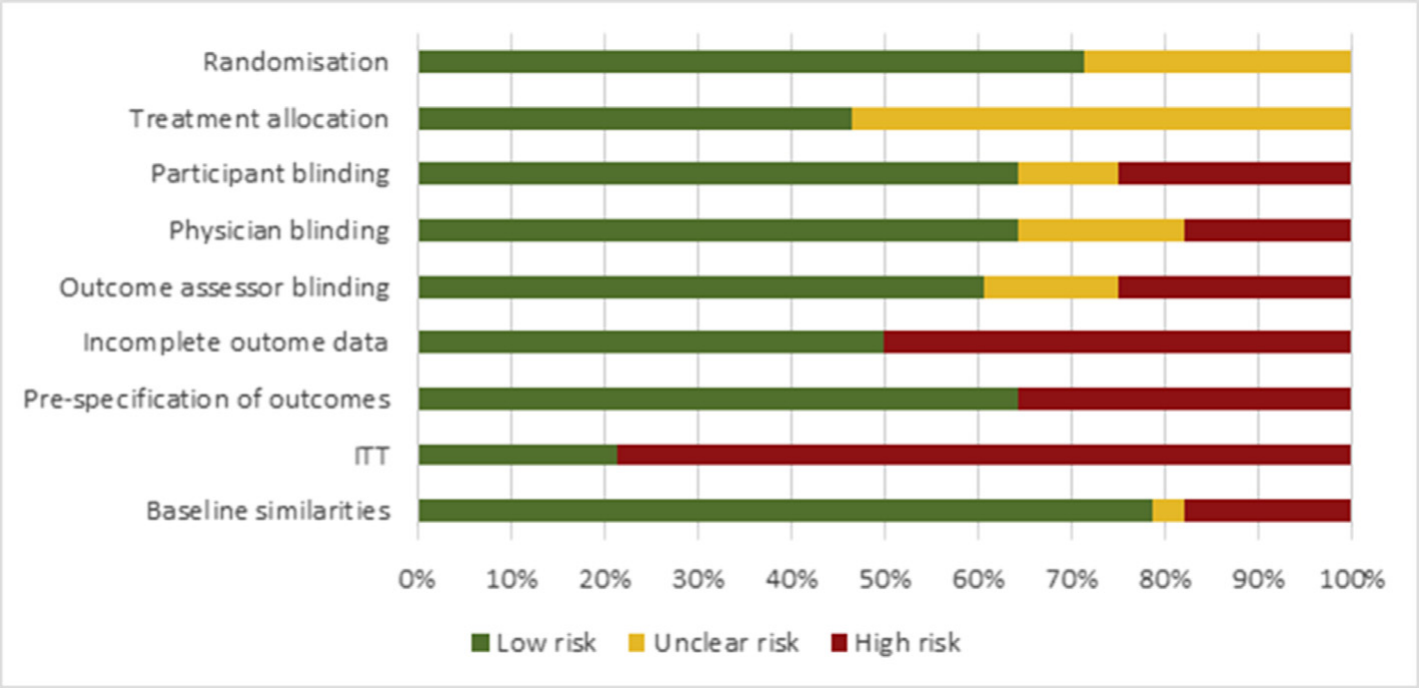
Risk of bias assessment. Risk of bias scores for all studies included in the overall analysis.

Trials were associated with considerable risks of bias (Fig. 2). Although described as randomised, only 20 publications described the method of random number sequence generation in sufficient detail to ascertain its risk of bias. Furthermore, only 13 of the included trials adequately described the methods of allocation concealment. Although described as double-blinded, this was only considered adequate in 60–65% of all trials. No capsaicin trials were deemed to adequately blind their participants due to the warming sensation experienced on its initial application. Across the body of evidence, only six of the 28 studies analysed all participants that were randomised at baseline.

### NMA

#### Overall analysis

**Fig. 3:**
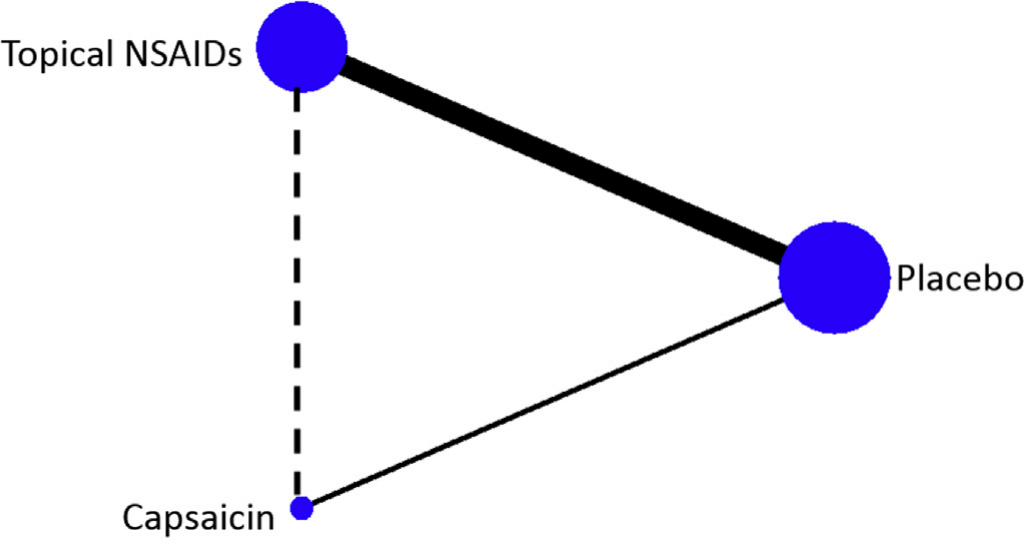
Trial network diagram. Nodes (circles) are weighted to represent the number of participants using each intervention. The solid lines represent the direct comparisons of the treatments in RCTs. The dotted line represents indirect comparisons generated through the NMA. The lines are weighted to represent the number of comparisons.

The trial network was comprised of 28 RCTs with 3473 participants on placebo (28 RCTs), 3693 on topical NSAIDs (23 RCTs), and 206 on capsaicin (5 RCTs) (Fig. 3). Direct evidence for topical NSAIDs vs placebo and capsaicin vs placebo were available from placebo-controlled trials. No trials directly compared topical NSAIDs to capsaicin, and the two treatments were therefore compared using placebo as a common comparator (indirect evidence).

Frequentist and Bayesian analyses were in agreement with identical ES and only minor differences in the CI vs CrI (Table I). Direct estimates indicated that topical NSAIDs were superior to placebo for pain relief. In contrast, the ES estimate between capsaicin and placebo was associated with considerable variability and did not reach statistical significance. However, the indirect analyses found no statistically significant differences between topical NSAIDs and capsaicin, although the ES favoured topical NSAIDs. Topical NSAIDs had the highest probability of being the best treatment, followed by capsaicin and then placebo (Table II).

**Table I:** ES and Frequentist CI/Bayesian CrI. Results of the overall and *as licensed* subgroup analysis of topical NSAIDs and capsaicin in OA

Comparison	Type	N	Frequentist		Bayesian	
			ES	CI	ES	CrI
**All trials**						
Topical NSAID vs placebo	Direct	23	0.30	0.19 to 0.41	0.30	0.19 to 0.43
Capsaicin vs placebo	Direct	5	0.27	−0.01 to 0.54	0.27	−0.02 to 0.56
Topical NSAIDs vs capsaicin	Indirect	28	0.04	−0.26 to 0.33	0.04	−0.28 to 0.35
***As licensed***						
Topical NSAID vs placebo	Direct	13	0.32	0.24 to 0.39	0.32	0.24 to 0.42
Capsaicin vs placebo	Direct	4	0.41	0.17 to 0.64	0.41	0.16 to 0.66
Topical NSAIDs vs capsaicin	Indirect	17	−0.09	−0.34 to 0.16	−0.09	−0.35 to 0.18

N: number of studies.

**Table II:** Treatment rankings. The probability of each treatment being the “best” using Frequentist and Bayesian approaches

	Probability of being the best (%)	
	Frequentist	Bayesian
**All trials**		
Topical NSAID	61.9	58.9
Capsaicin	38.1	41.1
Placebo	0.0	0.0
***As licensed***		
Topical NSAID	23.5	25.9
Capsaicin	76.5	74.1
Placebo	0.0	0.0

#### *As licensed* analysis

Topical NSAIDs and capsaicin were used as licensed in 17 RCTs. 1705 participants on placebo (17 RCTs), 1328 on topical NSAID (13 RCTs), and 141 on capsaicin (4 RCTs) were included in the *as licensed* NMA. The results are presented in Table I. Exclusion of non-licensed topical NSAIDs marginally raised the ES and it remained superior to placebo. In contrast, capsaicin at its licensed dose had a considerably increased ES that was statistically superior to placebo. Using placebo as a common comparator, no statistically significant differences remained between topical NSAIDs and capsaicin used as licensed. However, the ES favoured capsaicin, which also had the highest probability of being the best treatment, followed by topical NSAIDs and placebo (Table II).

## Discussion

Current evidence indicates that topical NSAIDs and capsaicin, when used as licensed, are both superior to placebo for pain relief. No significant differences were identified in the level of pain relief offered by topical NSAIDs compared to capsaicin. However, limited and poor quality evidence for capsaicin in OA provides uncertainty. Displaying seemingly negligible differences in efficacy, the decision of whether to prescribe topical NSAIDs or capsaicin should be guided by patient preference, safety, costs, and subsequent individual patient response.

Focussing on licensed doses of these two drugs renders the results of this meta-analysis more relevant for clinicians as they relate directly to the drugs recommended for prescription. The list of approved drugs was extracted from the BNF, a resource commonly used to guide prescribing practice in the UK^61^. The BNF was chosen as the leading authority on clinicians' selection of medicines in the UK, however it should be noted that they offer only recommendations of licensed medications and physicians can prescribe medications outside the recommended list^61^.

No direct or indirect (via NMA) quantitative evidence of the relative efficacy of topical NSAIDs vs capsaicin has been published previously. Some guidelines, such as those by Osteoarthritis Research Society International (OARSI) and European League Against Rheumatism (EULAR), provide equal recommendations for the two treatments^2^, ^4^, ^5^. This may indicate a perceived equivalence in efficacy, in line with the findings of the current meta-analysis. In contrast, a narrative review examining topical treatments in OA concluded that capsaicin had less efficacy than topical NSAIDs^62^. Similarly, topical NSAIDs are generally favoured in guidelines such as those by NICE and the American College of Rheumatology (ACR), perhaps indicating a postulated greater efficacy for topical NSAIDs^1^, ^3^. In addition, OARSI guidelines granted topical NSAIDs a greater mean benefit score (6.0/10) vs capsaicin (5.1/10)^2^. However, the comparative efficacy of the treatments in the narrative review was concluded primarily based on their mechanism of action, rather than quantitative analysis. Capsaicin was thought to be less effective as it lacked significant tissue penetration and anti-inflammatory effects^62^. Furthermore, guideline decisions are based not only on perceived efficacy, but on the quality of evidence. Indeed, the preference of topical NSAIDs may reflect a greater confidence in the evidence, rather than a perception of a larger effect. This is in keeping with the wide CI and associated uncertainty in the true effect of capsaicin in the current meta-analysis.

Although pain in OA has traditionally been viewed as nociceptive in nature, it is now widely accepted that some people experience pain with neuropathic-like pain components. Pain descriptors indicative of neuropathic pain, such as “burning” and “shooting” pain are used by subsets of individuals with OA^63^. In fact, almost 15% of people with knee pain report neuropathic-like pain^64^. This subgroup is of importance as true neuropathic pain is often difficult to manage and commonly does not respond to traditional analgesics, such as NSAIDs^65^, ^66^. Capsaicin, however, is licensed and used in neuropathic pain, where it is effective at higher doses^67^. It may therefore be that individuals with predominantly nociceptive OA pain benefit from topical NSAIDs whilst those with neuropathic pain components may benefit more from topical capsaicin. Further evidence on pain phenotypes and response to these two commonly used topical analgesics is warranted.

The present meta-analysis is subject to several limitations. Firstly, the conclusions drawn are limited by the scarcity of data available on capsaicin in OA. Only four trials compare 0.025% capsaicin to placebo and no direct estimates were available to compare topical NSAIDs to capsaicin. The low number of studies and participants on capsaicin resulted in an estimate with much uncertainty. The equivalence of the drugs may therefore be an artefact of the wide CIs. Secondly, the probability of being the best treatment is based predominantly on the ES, not on the uncertainty of the estimate. The probability of being the best was chosen to facilitate the translation of results to clinical practice, however the results should be interpreted with caution and in conjunction with the ES estimates. Thirdly, risk of bias assessment identified concerns over the high risk of bias in included trials. Poor compliance with complete outcome data reporting, analysis of all randomised participants, and pre-specification of published outcomes all have the potential to overestimate the results of this meta-analysis. Fourthly, because capsaicin is associated with a warming sensation on application, making it difficult to blind, it was deemed a high risk of bias domain for all capsaicin trials. This may result in inherent differences in the placebo group across the trial network, threatening the assumption of transitivity. Furthermore, the efficacy data for topical NSAIDs is predominantly based on knee OA (22 of 23 studies), whilst the trial population for capsaicin included hand, wrist, elbow, shoulder, hip, knee, and ankle OA. The differences in study populations may limit comparisons between the two treatments, however, it was not possible to conduct subgroup analyses by joint type due to limited data. Finally, by the very nature of analyses conducted at trial-level, the results of this NMA relate to populations of individuals with OA and may not be reflected at the individual patient level. In addition, data were unavailable to examine the efficacy of topical NSAIDs and capsaicin in subgroups with differing OA phenotypes (e.g., nociceptive vs neuropathic-like pain). Studies at the individual patient level are still required.

In conclusion, current evidence indicates that topical NSAIDs and capsaicin offer similar levels of pain relief in OA. Larger and better conducted RCTs, particularly for capsaicin, are required to confirm this. However, it is unknown whether individuals with different pain phenotypes respond differently to these two commonly used topical analgesics. Further work on phenotypic features of OA pain and their response to these two drugs is warranted.

## Author contributions

MSMP conceived the work, developed and ran the search strategy, screened trials for eligibility, designed data collection tools, performed data collection, analysed the data, and drafted and revised the paper. JS extracted data for validation and revised the paper. DAW, MD, and WZ were involved in the conceptualisation of the work, interpretation of the data, and revision of the paper. WZ is the guarantor. All authors discussed the results, commented on the manuscript, and have approved the final version of the paper.

## Conflicts of interest

MSMP and JS declare no support from any organisation for the submitted work; no financial relationships with any organisations that might have an interest in the submitted work in the previous 3 years; no other relationships or activities that could appear to have influenced the submitted work. DAW reports grants from Arthritis Research UK, during the conduct of the study; grants and personal fees from Pfizer Inc., personal fees from GSK Consumer Healthcare, outside the submitted work. MD reports grants from AstraZeneca funding a non-drug PI-led study in Nottingham (Sons of Gout Study), grants from Arthritis Research UK during the conduct of the study, and personal fees from Ad hoc Advisory Boards on osteoarthritis and gout for AstraZeneca, Grunenthal, Mallinckrodt and Roche, outside the submitted work. WZ grants from Arthritis Research UK Pain Centre, during the conduct of the study; personal fees from AstraZeneca and Hisun Pharm, royalties to his institution from EULAR, non-financial support from Peking University, Xiangya Hospital, outside the submitted work.

## Role of funding source

The work was supported by Arthritis Research UK [grant number 20777]. The funders had no role in study design, data collection, data synthesis, data interpretation, or writing the report.
