# Supplementary material for: The relative efficacy of topical non-steroidal anti-inflammatory drugs and capsaicin in osteoarthritis: a network meta-analysis of randomised controlled trials
Source: Osteoarthritis Cartilage. 2018 Dec;26(12):1575–82. doi: 10.1016/j.joca.2018.08.008 (PMC6267943; doi:10.1016/j.joca.2018.08.008)
Supplement: Multimedia component 2 [file mmc2.docx]

**Supplementary Information.** BNF recommended drugs for OA

| Drug | Concentration | Formulation |
| --- | --- | --- |
| Diclofenac diethylammonium | 1.16% | Gel |
| Felbinac |  | Gel |
| Ibuprofen | 5% or 10% | Gel |
| Ketoprofen | 2.5% | Gel |
| Piroxicam | 0.5% | Gel |
| Capsaicin | 0.025% | Cream |

* Equivalent to 1% diclofenac sodium^1^

1. eMC. Voltarol 1.16% Emulgel, gel - Summary of Product Characteristics. 2017 [cited 2017 28/11/2017]; Available from: <http://www.medicines.org.uk/emc/medicine/19985>.
