# Supplementary material for: The relative efficacy of topical non-steroidal anti-inflammatory drugs and capsaicin in osteoarthritis: a network meta-analysis of randomised controlled trials
Source: Osteoarthritis Cartilage. 2018 Dec;26(12):1575–82. doi: 10.1016/j.joca.2018.08.008 (PMC6267943; doi:10.1016/j.joca.2018.08.008)
Supplement: Multimedia component 3 [file mmc3.docx]

| **First Author** | **Publication Year** | **Joints Affected** | **Diagnosis Made Using** | **Treatment analysed (frequency per day)** | **Country of study** | **Funding source** | **Setting** | **Mean age (SD)** | **n (%) females** | **Week analysed** | **ES (seES)** | **Notes** |
| --- | --- | --- | --- | --- | --- | --- | --- | --- | --- | --- | --- | --- |
| Rother | 2013 | Knee | ACR | 2.29% ketoprofen gel (2) | USA | Commercial | Community | A: 61.8 (11.3), B: 62.6 (9.5) | 286 (52%) | 6 | -0.17 (0.09) |  |
| Conaghan | 2013 | Knee | ACR, Xray | 2.29% ketoprofen gel (2) | Czech Republic, Germany, Poland, UK | Commercial | Community | A: 61.6 (8),  B: 59.9 (9.5), C: 60.1 (9.8), D: 62.3 (8.2), E: 62.0 (8.7),  F: 61.3 (7.2) | 920 (66%) | 12 | 0.11 (0.06) |  |
| Altman | 2009 | Hand | ACR, Xray | 1% diclofenac sodium gel (4) | USA | Commercial | Unclear | A: 63.6 (10.3), B: 64.7 (9.6) | 296 (77%) | 4 | 0.27 (0.10) | AL |
| Simon | 2009 | Knee | Clinically, Pain, Xray | 1.5% diclofenac sodium solution (4) | Canada, USA | Commercial | Community | A: 61.7 (9.8), B: 62.1 (9.3), C: 61 (10),  D: 62 (10.5),  E: 60.6 (10) | 480 (62%) | 12 | 0.30 (0.10) | AL |
| Rother | 2007 | Knee | Other, Pain, PGA, Stiffness, Xray | 2.29% ketoprofen gel (2) | Germany | Commercial | Community | A: 63.3 (10.1), B: 62.4 (9.6), C: 62.8 (9.8) | 237 (60%) | 6 | 0.33 (0.12) |  |
| Niethard | 2005 | Knee | Clinically, Xray | 1.16% diclofenac diethylamine (4) | Germany | Commercial | Community | A: 66 (9),  B: 66 (9) | 151 (63%) | 3 | 0.36 (0.13) | AL |
| Baer | 2005 | Knee | Pain, Xray | 1.5% diclofenac sodium solution (4) | USA | Commercial | Community | A: 65 (11),  B: 64.6 (10.9) | 122 (56%) | 6 | 0.35 (0.14) | AL |
| Roth | 2004 | Knee | Pain, Xray | 1.5% diclofenac sodium solution (4) | USA | Commercial | Community | A: 63.4 (10.5), B: 64.9 (10.6) | 221 (68%) | 12 | 0.28 (0.11) | AL |
| Bookman | 2004 | Knee | Pain, Xray | 1.5% diclofenac sodium solution (4) | Canada | Commercial | Community | A: 62.5 (11.7), B: 62.1 (11.4), C: 60.8 (11.4) | 157 (63%) | 4 | 0.29 (0.14) | AL |
| Trnavsky | 2004 | Knee | ACR, Xray | 5% ibuprofen cream (3) | Czech Republic | Commercial | Community | A: 67 (6.7),  B: 66.9 (7.5) | 39 (78%) | 1 | 0.89 (0.30) | AL |
| Rovensky | 2001 | Knee | ACR, Xray | 5% ibuprofen cream (3) | Slovakia | Commercial | Community | A: 62.7 (7.3), B: 64.2 (9.1) | 74 (74%) | 1 | 0.84 (0.21) | AL |
| Ottillinger | 2001 | Knee | ACR, Xray | 0.1, 0.3, and 1% eltenac gel (3) | Hungary, Czech Republic, Switzerland, Germany | Commercial | Community | A: 66 (8),  B: 66 (9),  C: 67 (8),  D: 67 (7) | 183 (77%) | 4 | 0.10 (0.15) |  |
| Grace | 1999 | Knee | Symptomatic, Xray | 2% diclofenac sodium gel (3) | Canada | Both | Unclear | A: 60.4 (14.6), B: 63.6 (10.7) | 45 (61%) | 2 | 0.62 (0.25) | AL |
| Sandelin | 1997 | Knee | Pain, Xray | 1% eltenac gel (3) | Finland, Sweden | Unclear | Community | A: 61 (8.3),  B: 61 (7.9),  C: 61 (7.8) | 189 (65%) | 4 | -0.05 (0.14) |  |
| Baraf | 2010 | Knee | ACR, Xray | 1% diclofenac sodium gel (4) | USA | Commercial | Community | A: 61.8 (10.9), B: 60.9 (10.9) | 267 (64%) | 4 | 0.23 (0.10) | AL |
| Dreiser | 1993 | Knee | Pain, Xray | 180 mg diclofenac hydroxyethylpyrrolidine patch (2) | France | Unclear | Community | A: 66.8 (11.5), B: 64.8 (11.4) | 120 (77%) | 2 | 0.62 (0.17) |  |
| Yoo | 1996 | Knee | Clinically, Pain, Xray |  | Korea | Unclear | Community | A: 61 (7),  B: 59 (6) | 44 (100%) | 4 | 0.52 (0.37) |  |
| Bruhlmann | 2003 | Knee | Pain, Xray | 180 mg diclofenac epolamine patch (2) | Switzerland | Unclear | Community | A: 64 (10.7),  B: 64.8 (10.6) | 60 (58%) | 2 | 1.06 (0.21) |  |
| Varadi | 2013 | Knee | Pain, Symptomatic, Xray | 10% ibuprofen cream (2) | Switzerland, USA | Commercial | Community | A: 60.8 (11.6), B: 61.8 (11) | 48 (64%) | 2 | 0.25 (0.25) | AL |
| Kneer | 2013 | Knee | ACR, Xray | 2.29% ketoprofen gel (2) | Germany, Poland, Serbia, Croatia | Commercial | Community | A: 61.6 (9),  B: 61.9 (9.7), C: 61.8 (9.2), D: 61.3 (9.3) | 593 (68%) | 12 | 0.16 (0.08) |  |
| Shoara | 2015 | Knee | ACR, Xray | 1% diclofenac sodium gel (3) | Iran | Public | Community | A: 52.7 (7.4), B: 52 (8.9),  C: 50.6 (7.2) | 71 (72%) | 3 | 0.28 (0.27) | AL |
| Wadsworth | 2016 | Knee | Xray | 2% diclofenac sodium gel (2) | USA | Commercial | Community | A: 60.2 (9.2), B: 61.9 (9.1) | 273 (72%) | 4 | 0.21 (0.13) | AL |
| Yataba | 2017 | Knee | Xray | 10, 20, 40 mg S-flurbiprofen patch (1) | Japan | Commercial | Community | A: 66.7 (9.6), B: 66.4 (9.3), C: 66.2 (8.6), D: 67.3 (9.1), E: 66.4 (9.1) | 654 (85% | 2 | 0.27 (0.10) |  |
| Kosuwon | 2010 | Knee | ACR, Xray | 0.0125% capsaicin cream (3) | Thailand | Both | Community | 61 (6.3) | 99 (100%) | 4 | -0.18 (0.18) |  |
| McCleane | 2000 | Hand, Hip, Knee, Shoulder | Xray | 0.025% capsaicin cream (4) | UK | Commercial | Community | A: 49.7 (13.4), B: 48.4 (14.1), C: 48.1 (14.3), D: 50.9 (12.8) | 89 (45%) | 4 | 0.18 (0.22) | AL |
| Schnitzer | 1994 | Hand | Clinically, Xray | 0.025% capsaicin cream (4, then 2) | USA | Commercial | Unclear | A: 69.3 (8.4), B: 66.8 (9) | 40 (68%) | 3 | 0.78 (0.32) | AL |
| Altman | 1994 | Ankle, Elbow, Knee, Shoulder, Wrist | Clinically, Pain, Xray | 0.025% capsaicin cream (4) | USA | Commercial | Community | A: 63 (12),  B: 61 (12) | 72 (64%) | 4 | 0.46 (0.20) | AL |
| Deal | 1991 | Knee | Clinically, Xray | 0.025% capsaicin cream (4) | USA | Public | Community | A: 62 (10.8),  B: 60 (11.8) | 45 (64%) | 4 | 0.38 (0.25) | AL |
| ACR: American College of Rheumatology; AL: As Licensed analysis; ES: effect size; seES: standard error of effect size; n: number; PGA: Patient/Physician Global Assessment  Mean age (SD) presented for all treatment arms in study, listed as A, B, C, etc. | | | | | | | | | | | | |
